# Can the accepting foreign clinical data policy improve innovation investment of pharmaceutical firms? Empirical evidence from China

**DOI:** 10.3389/fpubh.2025.1512148

**Published:** 2025-07-30

**Authors:** Mengjuan Jiang, Jingjing Huang, Su Wang, Yulu Fan, Yuwen Chen

**Affiliations:** ^1^School of Business Administration, Shenyang Pharmaceutical University, Shenyang, China; ^2^College of Pharmacy, Shanghai University of Medicine & Health Sciences, Shanghai, China; ^3^Research Institute of Drug Regulatory Science, Shenyang Pharmaceutical University, Shenyang, China

**Keywords:** foreign clinical data, innovation investment, difference-in-differences model, pharmaceutical firms, absorptive capacity

## Abstract

**Introduction:**

In October 2017, China initiated adjustments to the registration procedures for imported drugs to promote accessibility to overseas drugs. In support of this decision, the National Medical Products Administration (NMPA) issued a technical guideline regarding accepting foreign clinical trial data in July 2018. Collectively referred to as the accepting foreign clinical data policy, these measures have accelerated the influx of overseas drugs into China.

**Methods:**

Using the panel data from 104 A-share pharmaceutical listed firms between 2013 and 2024, this study conducted a difference-in-differences model to explore the impact of this policy on the innovation investment of Chinese pharmaceutical firms and further analyzed the underlying moderating effect.

**Results:**

The results demonstrate a significantly positive effect of this accepting foreign clinical data policy on pharmaceutical firms’ innovation investment, which is verified with the parallel trend and robustness tests. Further analysis indicates that corporate absorptive capacity positively moderates the relationship between the policy implementation and innovation investment. In addition, the heterogeneity analysis suggests that this policy has a more significant effect on firms that are non-state-owned, engage in new drug research and have strong market power.

**Discussion:**

This study serves as a significant supplement to the current literature regarding the accepting foreign clinical data policy and innovation investment, providing valuable insights for policymakers and R&D decision-makers in the pharmaceutical sector.

## 1 Introduction

Clinical trials are the most time-consuming and expensive steps during the research and development (R&D) process of new drugs, which provide substantial evidence to evaluate the efficacy and safety of drugs for the registration and approval of drugs (1). With the accelerating globalization of pharmaceutical clinical research and development, more and more pharmaceutical firms are conducting international multi-regional clinical trials (MRCTs) to support global registration applications, so as to improve R&D efficiency, reduce costs and unnecessary redundant studies, and expand market shares (2). However, the simultaneous conduct of clinical trials for new drugs worldwide and the acceptance of foreign clinical data is often hampered by differences in drug registration and clinical trial administration systems between countries or regions. Moreover, different national regulatory authorities have different understandings of foreign clinical data due to differences in technical review requirements, ethnic populations of the subjects, clinical trial conditions and other factors affecting clinical trial outcomes. These differences make it complicated and controversial for them to accept foreign clinical trial data for marketing applications of overseas drugs (3).

In the United States, according to Title 21 of the Code of Federal Regulations, Part 314.106, regulations since 1985 have explicitly contemplated reliance on non-US data as part of, or even as the entire basis for drug approval, provided that the foreign data are applicable to the US population and medical practice (2). In Europe, it was reported as early as 1988 that the quality of clinical data, regardless of its origin, was the determining factor for marketing authorization in the European Community and other countries (4). In Japan, the earliest country in Asia to introduce the International Conference on Harmonization E5 (ICH-E5) in 1998, simultaneous conduct of clinical trials for foreign new drugs was permitted. However, due to the differences in cultural and ethnic factors, as well as relatively strict and conservative supervision, additional local clinical trials were necessary to meet Japanese regulatory requirements (3). With the globalization of clinical research and the accumulation of ethnic data, regulatory authorities are progressively adopting new scientific evaluation methodologies and exhibiting a more open stance toward accepting foreign clinical data.

**Figure 1:**
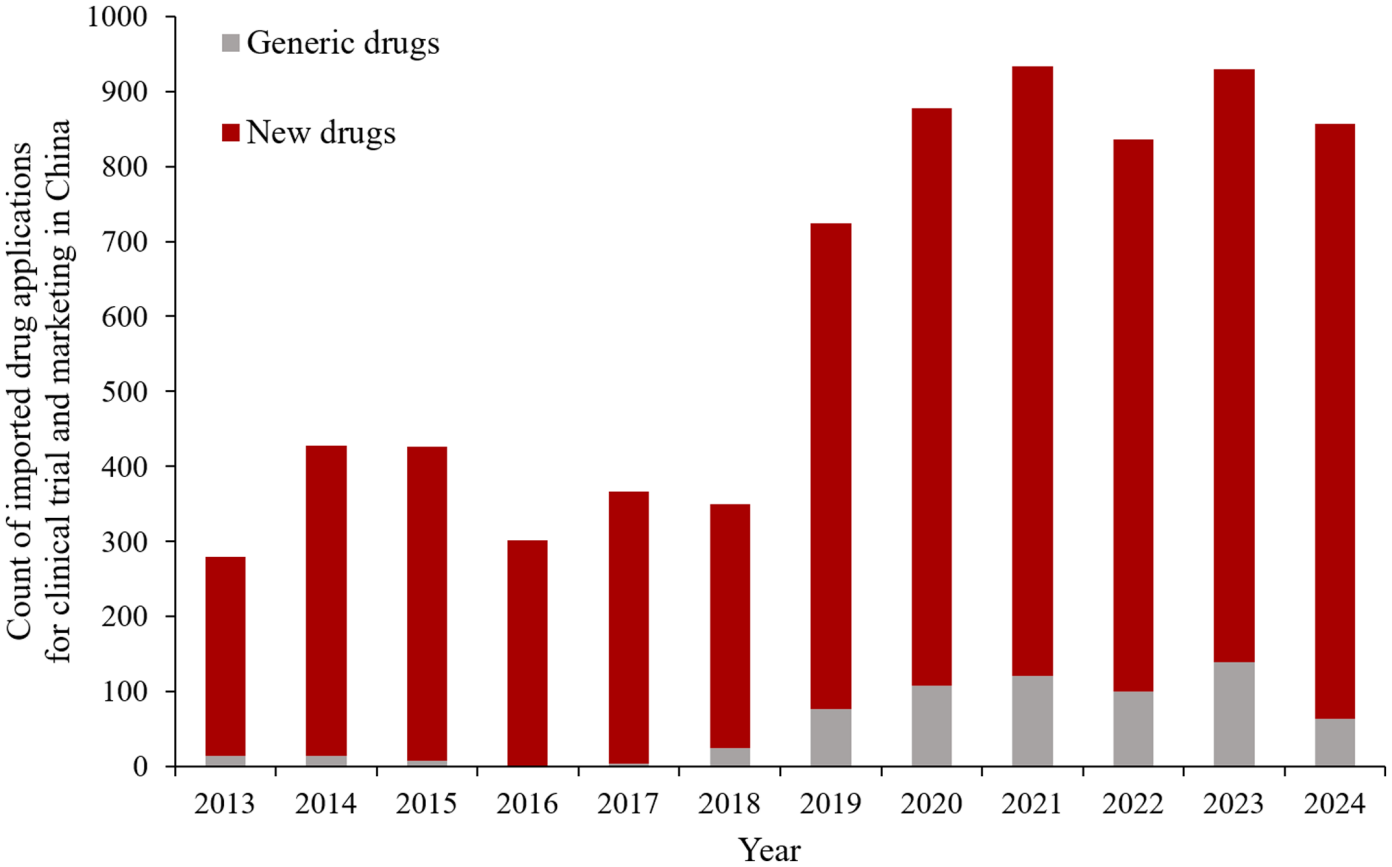
The count of imported drug applications for clinical trial and marketing in China. *Data from Yao Zhi, https://db.yaozh.com/.

In China, the regulatory authority applied stringent requirements for initiation of clinical trials and acceptance of foreign clinical data in the past (5). Clinical trials were not allowed to be simultaneously conducted in China and abroad, and supplementary clinical development programs specifically involving Chinese patients were compulsory (6). It was not until October 2017 that the Chinese regulatory authority abolished the requirements that a new drug must have initiated at least a phase 2 trial overseas before Chinese sites participate in the MRCT and must obtain marketing authorization from other countries before applying for approval in China (6–8). In addition, marketing applications can be submitted directly for overseas drugs with MRCTs containing Chinese data. Subsequently, in order to further encourage the simultaneous R&D process and expedite the marketing process of overseas new drugs in China, the regulatory authority issued the guidance on accepting foreign clinical trial data and established a special approval channel for urgently needed overseas new drugs in 2018 (9, 10). Collectively referred to as “accepting foreign clinical data” in this paper, these measures encourage a shift in the R&D strategy of imported drugs toward participation in MRCTs in China prior to approval, resulting in an increase in the number of MRCTs conducted in China (7, 11). In addition, the approval time for imported drugs has been significantly shortened (12). As a result, the policy of accepting foreign clinical data has encouraged the registration applications of overseas drugs and accelerated their entry in China. As shown in Figure 1, the number of clinical trial and marketing applications for imported drugs has increased by two to three times since 2018. Consequently, the accepting foreign clinical data policy has undoubtedly enhanced the import competition in Chinese pharmaceutical industry.

Continuous innovation consistently serves as the pivotal source of competitive advantage in the pharmaceutical sector (13). To enhance the innovation capabilities of pharmaceutical firms and meet the evolving clinical needs of residents, the Chinese government has implemented a series of reforms and policies in this field, including consistency evaluations of generic drugs (14, 15), volume-based procurement (16), price negotiation in medical insurance (17), and the Marketing Authorization Holder system (13). Empirical evidence has verified that these policies effectively stimulate corporate innovation. The policy of accepting foreign clinical data, which is a key reform in the drug registration system, facilitates the influx of overseas drugs and subsequently increases the import competition. As there is still a significant gap between Chinese firms and international experienced firms in terms of innovation capabilities, funds, talents and management systems (17), this policy will have a great impact on the innovation incentives of Chinese pharmaceutical firms due to intensified import shock. Some industry scholars argue that this policy will put tremendous pressure on domestic firms and discourage their further innovative efforts, while others believe that it will force domestic firms to invest more in innovation to improve their competitiveness (18). To date, studies on the relationship between the accepting foreign clinical data policy and pharmaceutical firms’ innovation is lacking and no consensus has been reached.

To address the aforementioned questions, we conduct a theoretical analysis of how the accepting foreign clinical data policy influences innovation investment of Chinese pharmaceutical firms based on the marketing competition effect and technological spillover effect. Subsequently, this paper considers this policy as a quasi-natural experiment and takes 104 A-share listed pharmaceutical firms from 2013 to 2024 as the research samples for empirical verification by a difference-in-differences (DID) model. Furthermore, given that absorptive capacity can influence corporate innovation in extant research (19), we also examine the moderating role of corporate absorptive capacity in the relationship between this policy and innovation investment. In summary, the objective of this study is to validate whether the implementation of the accepting clinical foreign policy in China can effectively stimulate the innovation incentives among pharmaceutical firms and to explore its underlying influencing mechanism. This study serves as a significant supplement and expansion to the current literature regarding the accepting foreign clinical data policy and innovation, providing crucial empirical support for the further enhancement of the policies to encourage the launch of overseas drugs. It also offers valuable insights for policymakers and R&D decision-makers in the pharmaceutical sector.

## 2 Institutional background

In China, prior to 2000, overseas drugs with unmet medical demands could be approved without requiring additional clinical trials conducted in China. With the accumulation of R&D and regulatory experience, the regulatory authority began to require Chinese clinical data before marketing approval (7). According to the China Drug Registration Regulation (DRR), a core document governing clinical trials and drug registration issued in 2007, the global pharmaceutical sponsors were required to conduct an additional standalone clinical development program containing pharmacokinetic studies and at least 100 pairs of randomized controlled clinical trials specifically in Chinese patients, even though their drugs had been approved overseas (5, 6). In addition, for overseas new drugs, initiation of phase II/III clinical trials overseas was required for Chinese sites to participate in the MRCTs, and holding marketing authorization in other countries was a prerequisite for applying for marketing in China (5). These local requirements have posed a significant obstacle to approval process of overseas drugs in China. It was reported that during the period from 2004 to 2014, among the 291 new molecular entities approved by the US FDA, only 79 (27%) were approved in China with an average delay of 3 years compared to those in THE US (20). The lag in drug approval has hampered the marketing of overseas drugs and significantly limited their accessibility in China (6).

The above regulations concerning imported drugs in China were not amended for the following decade until 2015. From this year, China initiated a deep reform on the drug review and approval process. The landmark documents of “Opinions of the State Council on Reforming the Review and Approval System for Drugs and Medical Devices” [State Council (2015) No.44] released in August 2015 (21) and “Opinions on Deepening the Reform of Review and Approval System to Encourage Innovation of Drugs and Medical Devices” [State Council (2017) No.42] released in October 2017 (22) proposed to allow simultaneous clinical trials conducted in China and to accept foreign clinical trial data for overseas drugs. In response to these opinions, to facilitate the launch of overseas new drugs and improve patients’ accessibility to unmet clinical demand, the former China Food and Drug Administration (CFDA) issued the document “Decisions on the adjustment of imported drug registration [CFDA (2017) No.35]” in October 2017 (8). This document officially permits that early phase clinical trials of imported new drugs can be conducted simultaneously in China and abroad, and abolishes the requirement that imported drugs must obtain marketing authorization in other countries before applying for marketing. In addition, overseas drugs with MRCTs containing Chinese data can apply directly for marketing with the wavier of clinical trial applications. However, this document did not formulate specific implementation measures on how to accept foreign clinical data. Subsequently, in July 2018, the technical guideline on accepting foreign clinical data were formally issued by the National Medical Products Administration (NMPA), which explicitly specifies the principles and requirements on how to accept foreign data, as well as the acceptability of foreign clinical data (9). The guideline emphasizes that depending on the quality, completeness and ethnic sensitivity of the foreign clinical data, the authority may accept the data fully, partially or not at all. In addition, foreign clinical data can be accepted not only from overseas new drugs, but also from overseas generics and biosimilars. In October 2018, the Chinese authority established special approval channels to expedite the review of clinically urgent needed overseas drugs (UNOD) (10). The expedited approval of imported drugs with limited or even no Chinese clinical data if no ethnic differences on the UNOD lists released between 2018 and 2020 illustrates canonically accepting foreign data when recognized with the unmet needs and drug delay in treating severe or life-threatening diseases (23).

## 3 Literature review

### 3.1 Studies on the accepting foreign clinical data policy

The current studies on the policy of accepting foreign clinical data have mainly focused on the requirements for foreign data quality, ethnic differences and bridging trials. For example, Harpreet Singh and Richard Pazdur explored the acceptance and challenges of new drug applications based on Chinese clinical trial data in the US, including ethnic representation, comparator therapies and occasional data integrity issues (24). Chang et al. discussed the considerations of ethnic differences and bridging trials for accepting foreign clinical data in the US, EU, Japan, and China (25). In addition, a limited number of scholars have examined the implementation effect of the policy on the Chinese pharmaceutical industry, specifically focusing on aspects such as the quality of clinical trials and the approval speed of new drugs. Jiang et al. posited that this policy not only has the potential to enhance clinical data standards in accordance with international benchmarks but also reinforces Chinese pharmaceutical firms’ emphasis on clinical trial data management (26). Liu et al. believed that the proportion of drugs adopting the MRCT pathway has begun to expand and the gap in drug approval between China and other countries has been remarkably shortened due to the implementation of this policy (7). Luo et al. proposed that the accepting clinical trial data for overseas drugs has greatly boosted enthusiasm for innovative drug development. The initiative has substantially increased the number of IND applications and accelerated the approval process of innovative drugs for marketing in China (12).

Through the systematic review of the literature, it is found that existing academic studies on examining the impact of this policy on innovation within pharmaceutical firms are scarce, especially in terms of quantitative analysis. This gap presents an opportunity for potential contributions.

### 3.2 The relationship between the accepting foreign clinical data policy and corporate innovation investment

There is a limited number of studies on the impact of the accepting foreign clinical data policy on corporate innovation. Since this policy can promote influx of overseas drugs into China (7, 12), which suggests the influence of this policy on the innovation investment of domestic pharmaceutical firms can be theoretically analyzed through the lens of import competition. In this study, we propose that this policy may affect innovation investment through two primary mechanisms: the marketing competition effect and the technological spillover effect.

As for the marketing competition effect, Schumpeterian growth theory holds that economic growth is primarily driven by quality-improving innovations, and the pursuit of monopoly rents by enterprises serves as a significant incentive for innovation (27). Competition plays a crucial role in innovation activities as it has the potential to diminish monopoly rents (28). On one hand, according to Schumpeter’s appropriability argument, import competition diminishes post-innovation rents, such as market share and production scale, thereby reducing ex-ante innovation incentives (27, 29). Empirical evidence supporting this perspective can be found in studies such as those conducted by Autor et al. (30), who discovered that rising competition from imports has compelled American firms to reduce operational expenses in R&D, consequently hindering their innovation activities. Similarly, Liu et al. (31) demonstrated that import competition adversely affects innovation in the Chinese manufacturing sector based on the data on industry tariffs and firm patents. On the other hand, due to heterogeneity among enterprises, industries, and regions, in fact, import competition can also stimulate domestic firms to enhance product differentiation and quality by innovation behaviors for retaining or expanding their market shares (32). This phenomenon, commonly referred to as the escape-competition effect, has found support in numerous empirical studies (33–35). For instance, Bombardini et al. found top firms featured increased R&D expenditures and an increase in market shares following import liberalization (33). Bloom et al. argued that import competition could improve the level of patent output and R&D investment of European countries by freeing up the labor and lowering the opportunity cost of firms to switch technology (36).

In China, the policy of accepting foreign clinical data relaxes the registration restrictions of imported drugs, thereby encouraging international pharmaceutical firms to incorporate China in their global registration strategies and facilitating an accelerated influx of overseas drugs into China (7). Consequently, domestic pharmaceutical firms have to face a stiffer competition from imported drugs. Over the past few decades, Chinese innovation in new drug development has predominantly relied on modifying the molecular structures of foreign original new drugs with existing or potential therapeutic targets and mechanisms without infringing patents, which is commonly called “fast-follow” or imitative innovation (37). The registration barrier for imported drugs led to a prolonged drug lag in approval between China and foreign manufacturing countries, which has greatly benefited Chinese pharmaceutical firms due to the low competition from imported drugs. However, an accelerated influx of overseas drugs into China is narrowing the window of drug lag, which not only poses increasing challenges for domestic new drugs with the “fast-follow” innovation strategy to maintain their pioneer advantages, but also makes it become difficult for domestic new drugs with similar therapeutic targets and indications to imported drugs to qualify for expedited review (18, 38). In recent years, the development of China’s pharmaceutical industry has been advancing rapidly under the government guidance, and the potential market opportunities remain substantial (39). To create new market opportunities and reshape their own competitive advantages, Chinese pharmaceutical firms will be compelled by this policy to increase their investment in enhancing the innovation capacity to explore first-in-class drugs or develop new technologies for me-better drugs.

Additionally, regarding the technological spillover effect, it refers to the transmission of knowledge and technological learning that enables latecomers to benefit from the R&D efforts of their peers in closely related technological fields (40, 41). The technological gap theory posits that international disparities in technological capabilities serve as a primary driver of cross-border technology diffusion, wherein less developed countries learn from and imitate advanced technologies from technologically superior countries through trade, thereby catalyzing technological convergence (41). Consequently, imports activities act as a significant channel for domestic firms to access not only tangible technological inputs but also embedded tacit knowledge (42). Domestic industries are able to learn from and assimilate imported advanced technologies, including product processes, designs, and managerial practices, to enhance their innovation capacity and improve their competitiveness through the technological spillover effect (43). Furthermore, they can rapidly accumulate both technology and human capital by reverse-engineering foreign technologies and introducing relative talents, ultimately reducing trial-and-error costs and increasing R&D efficiency (44). A body of literature has demonstrated that import-related technological spillovers, which allow domestic firms to learn and assimilate new technologies from their foreign counterparts, can promote domestic technological innovation (42, 45, 46). Firms will increase their investment in R&D expenditures to effectively absorb and transform these technological spillovers (47). Wang et al. found that the international R&D spillover through inward FDI and imports significantly enhances China’s innovation performance (46). Sun et al. showed with the horizontal or mixed cooperation, corporate R&D investment increases as horizontal spillovers rise (48).

The implementation of accepting foreign clinical data supports China’s early participation in MRCTs and paves the way for the development and registration of imported drugs in China. Consequently, there has been a significant increase in the number of imported drugs adopting the MRCT pathway in China (7). On one hand, the increase in MRCTs facilitates the diffusion of advanced clinical technologies (such as innovative study methodologies, digital technologies, electronic equipment), talents and management systems from leading global pharmaceutical firms into China. Chinese pharmaceutical firms have long been criticized for their limited capabilities of clinical studies and poor quality of clinical trials (49). To enhance their R&D efficiency and meet escalating standards for clinical trials, the diffusion will drive increased investment in introducing skilled clinical teams, as well as acquiring advanced technologies and equipment (26). On the other hand, simultaneous or early-stage clinical studies of overseas new drugs conducted in China allow domestic firms to gain valuable insights into cutting-edge product innovations, including therapeutic targets, indications and clinical outcomes. Chinese pharmaceutical firms are transitioning from producing generic drugs to developing innovative drugs (39). To improve their innovative capacity and efficiency, they will increase R&D investment in following these new drugs or exploring first-in-class drugs based on these new therapeutic mechanisms. Additionally, the accelerated listing of original imported drugs enhances access to reference preparations in China, thereby facilitating the development of generic drugs, which can also be regarded as a form of technological spillover. Consequently, there will be an increased investment in the consistency evaluation for generic drugs (14). Based on the above analysis, we propose the following hypothesis:

*H1*: The policy of accepting foreign clinical data can promote the innovation investment of pharmaceutical firms.

### 3.3 The moderating effect of absorptive capacity

Absorptive capacity is defined as the ability of an enterprise to recognize the value of new and external information, assimilate it, and apply it to commercial ends (50). As external knowledge serves as a vital source of innovation, absorptive capacity is considered as one of the most significant determinants of a firm’s ability to enhance the innovation performance (51). By leveraging the absorptive capacity, firms not only expand their knowledge reservoirs and skill base but also improve their ability to assimilate and operationalize emerging information, ultimately driving technological advancements and enhance innovation outcomes (52). This assertion has been substantiated by numerous previous empirical studies. Sikka argued that a high level of absorptive capacity enhances corporate R&D capabilities, thereby leading to improved corporate innovations (53). Zou et al. posited that a positive relationship exists between corporate absorptive capacity and their innovation performance by using a meta-analysis, and this relationship is more pronounced in the context of incremental innovation compared to radical innovation (54).

Moreover, in order to enhance absorptive capacity and effectively assimilate external knowledge, firms must allocate resources and invest internally, particularly in research and development activities (50). Consequently, absorptive capacity also plays a positive moderating effect on firms’ R&D investment. For instance, Veugelers discovered that the external sourcing of knowledge will enhance internal R&D investment only if a firm maintains an adequate level of absorptive capacity (55).

In this study, while the accepting foreign clinical data policy may incentivize pharmaceutical firms to innovate, it is also crucial for them to possess sufficient absorptive capacity to value, assimilate and transform external new knowledge and technologies to innovation outcomes (50). Based on the previous review, firms’ absorptive capacity is positively correlated with their innovation performance. Enhanced capabilities in assimilating external knowledge not only predictably improve technological outcomes but also strengthen innovation incentives by demonstrating the tangible returns on R&D investments. Consequently, faced with the accelerated influx of imported drugs, enterprises with strong absorptive capacity will be more inclined to adopt a proactive innovation strategy than those firms with a low level of absorptive capacity. Furthermore, an enterprise with higher absorptive capacity can achieve greater product effectiveness and market value due to its abundance of resources, extensive experiences and diverse R&D activities (43). The desire to assimilate external know-how creates a positive incentive to invest in R&D (55). Thus, the higher the level of knowledge absorptive capacity, the more innovation activities can be stimulated. Additionally, absorptive capacity can partially mitigate the uncertainty caused by the policy changes to some extent, maintain the confidence of firms in R&D and alleviate potential negative effects on firms’ innovation investment (56). Based on the above discussion, we expect that the positive effect of the policy of accepting foreign clinical data on corporate innovation investment can be enhanced by corporate absorptive capacity. Therefore, the following hypothesis is proposed:

*H2*: Absorptive capacity positively moderates the effect of the accepting foreign clinical data policy on innovation investment.

**Figure 2:**
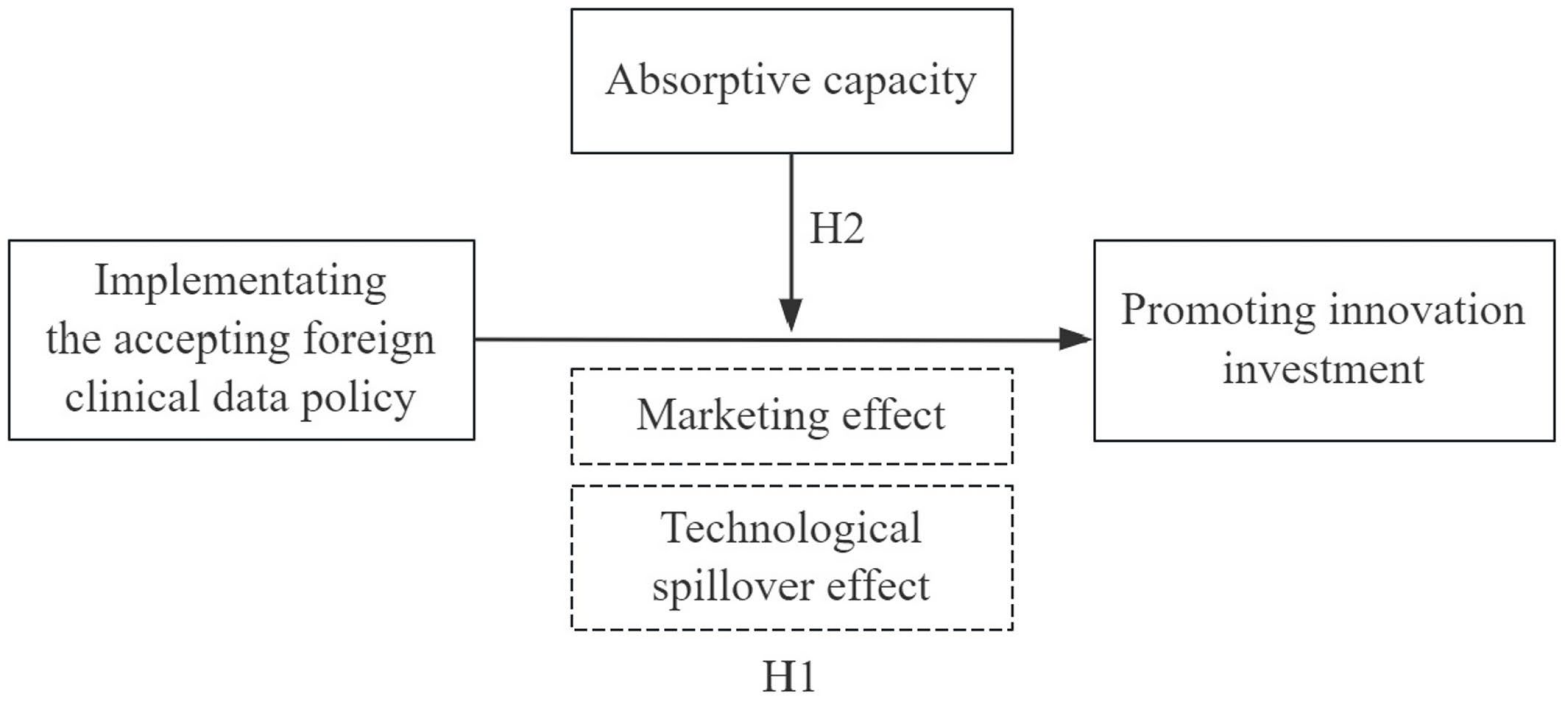
Conceptual framework diagram.

The conceptual framework hypothesized in this study is depicted in Figure 2.

## 4 Research design

### 4.1 Sample selection and data sources

Based on the industry classification standard system issued by Shenyin & Wanguo Securities Co., Ltd. in July 2021, we take the firms of chemical pharmacy, traditional Chinese medicine and biologics sub-sectors under the pharmaceutical biology sector listed on the A-share market as the research samples. The main commercial activities of pharmaceutical firms in the other three sub-sectors are pharmaceutical trade, medical devices, and medical services (14). As this study aims to explore the impact of the accepting foreign clinical data policy on the innovation investment of pharmaceutical manufacturers, these samples in these three sub-sectors are excluded. The raw data is collected from the China Stock Market Accounting Research Database (CSMAR, an authoritative database from China) and is further processed as the following principles: (1): firms marked with ST or ^∗^ST are excluded, which are subject to delisting risk warnings; (2) firms with incomplete disclosure of primary data and related indicators are excluded; (3) firms with abnormal data are excluded; (4) firms listed after December 31, 2013 are excluded; (5) In order to mitigate the impact of outliers, all the continuous variables are dealt with by replacing the top and bottom 1% with the value of the observation at 99 and 1% levels. Finally, we obtained the panel data of 104 A-share pharmaceutical listed firms during the period of 2013 ~ 2024 for the empirical analysis, with a total of 1,248 observations.

### 4.2 Variables

#### 4.2.1 Explained variable

Corporate innovation investment, or R&D investment intensity, is used extensively as a proxy for innovation (13). The higher a firm’s innovation investment, the stronger its strategic focus is on innovation, since that the firm has chosen to invest a higher percentage of turnover in innovation activities. Based on previous literature and data availability, we select innovation investment as the explained variable, which is calculated by dividing R&D expenditures by the firm’s operating revenue to reduce the impact of firm size on the proxy (13, 14).

#### 4.2.2 Explanatory variable

In October 2017, the CFDA issued the document of “Decisions on the adjustment of imported drug registration [CFDA (2017) No. 35]” to allow simultaneous first-in-human studies tested in Chinese participants for overseas new drugs and direct marketing applications with the MRCTs containing Chinese data, and to remove the requirement that imported drugs must have obtained the marketing approval in other countries before applying for marketing in China, which meant that the Chinese regulatory authority began to accept the clinical trial data conducted outside China (8). Subsequently, in July 2018, the NMPA released the guideline to formally confirm the principles and requirements for accepting foreign clinical trial data (9). Therefore, this study selects the year 2018 as the time point of policy implementation, with Post denoting the time dummy variable assigned a value of 1 if Post ≥ 2018 and 0 if Post < 2018.

The acceptance of foreign clinical data relaxes the registration restriction to some extent, which has resulted in an increased rate of registration applications for imported drugs in China. This policy particularly affects the firms engaging in the research of new drugs with the same targets and indications as the imported new drugs, as well as those involved in developing generic drugs or biosimilars of imported drugs (18, 38). Therefore, we sorted out the firms mentioned above as the treatment group from the Drugdataexpy (Chinese phonetic alphabet: Yao Zhi) database built by Chongqing Kangzhou Big Data Co., Ltd., one of the most authoritative platforms providing extensive data services and empowerment in China’s medical and health industry, and the remaining firms as the control group. Treat represents the group dummy variable, which equals 1 when the firm is in the treatment group, or 0 when the firm is in the control group.

The final core explanatory variable Treat×Post is measured by the interaction of the treatment group dummy variable Treat and time dummy variable Post to measure the net impact of this policy.

#### 4.2.3 Control variables

To mitigate the influence of factors other than the core explanatory variable on the explained variable, this study draws upon previous research (13, 14) and selects seven firm-level control variables based on several key dimensions: financial characteristics (asset-liability ratio, cash flow ratio), governance structure (ownership concentration), individual characteristics (size, age) and operating conditions (return on equity, firm’s growth ability).

#### 4.2.4 Moderating variable

The accumulation of knowledge and talent is a critical determinant of firms’ absorptive capacity (50). Referring to the aforementioned literature (57), we select the proportion of R&D personnel as a measure of absorptive capacity.

The specific definitions of each variable are shown in Table 1.

**Table 1:** Definition and calculation method of main variables.

Variable type	Variable name	Variable definition	Variable symbol
Explained variable	Innovation investment	R&D expenditures / Operating income (%)	Input
Explanatory variable	Treat×Post	The interaction term of the group dummy variable and the time dummy variable	DID
Moderating variable	Absorptive capacity	The ratio of R&D personnel (%)	AC
Control variables	Firm size	Ln (total assets)	Size
	Asset-liability ratio	Total debt / Total assets (%)	Lev
	Cash flow ratio	Cash from operating activities / Total assets (%)	Cash
	Return on equity	Net profit / Stockholders’ equity balance (%)	Roe
	Firm’s growth ability	Operating income growth rate (%)	Growth
	Ownership concentration	The shareholding ratio of the top 10 major shareholder (%)	Shareh10
	Firm’s age	Ln (Sample period-establishment period+1)	Age

### 4.3 Equation design

The DID model can effectively mitigate endogeneity problems among heterogeneous individuals to a large extent by capturing the changes in outcome variables before and after policy implementation (58). In this study, we treat the policy of accepting foreign clinical data as an exogenous shock and construct a DID model to investigate the impact of this policy on innovation investment of pharmaceutical firms. The baseline regression model is specified as follows:


(1) $$\text{Inpu}t_{\mathrm{it}}=\alpha +\text{βTrea}t_{\mathrm{it}}\times \mathrm{Pos}t_{\mathrm{it}}+\theta X_{\mathrm{it}}+\lambda_{i}+\upsilon_{t}+\varepsilon_{\mathrm{it}}$$

In Equation 1, $\text{Inpu}t_{\mathrm{it}}$ denotes the innovation investment of pharmaceutical firm i in year t . The effect of the accepting foreign clinical data policy is measured by the interaction term $\text{Trea}t_{\mathrm{it}}\times \mathrm{Pos}t_{\mathrm{it}}$ . A significantly positive estimated coefficient β suggests a positive policy effect on the innovation investment of listed pharmaceutical firms, while a significantly negative coefficient β indicates a negative policy effect. $X_{\mathrm{it}}$ represents all the control variables, $\varepsilon_{\mathrm{it}}\;$ denotes a random error term, $\lambda_{i}$ represents individual fixed effects and $\upsilon_{t}$ denotes time fixed effects.

## 5 Results

### 5.1 Descriptive statistics

Table 2 reports the descriptive statistical findings of the main variables in this study. It can be seen that the innovation investment of pharmaceutical firms in China varies widely, with a minimum value of 0.019, a maximum value of 123.019, and an overall mean of 6.019. This indicates that the innovation investment of Chinese pharmaceutical firms remains at a relatively low level and needs to be further improved. In terms of control variables, according to the mean and standard deviation (SD), the differences in Size and Age among different pharmaceutical firms are minor, while Lev, Cash, Roe, Growth and Shareh10 exhibit significant fluctuations and considerable variability.

**Table 2:** Descriptive statistical results.

Variable	Obs	Mean	SD	Min	Median	Max
Input	1,248	6.019	7.167	0.019	4.289	123.019
Size	1,248	22.373	1.003	19.391	22.285	25.489
Lev	1,248	32.404	18.149	1.427	30.134	90.343
Cash	1,248	6.629	6.069	−23.774	6.231	47.063
Roe	1,248	5.985	25.270	−580.143	7.717	66.431
Growth	1,248	11.160	31.351	−65.700	9.255	503.369
Shareh10	1,248	53.478	14.898	18.850	53.003	91.927
Age	1,248	3.044	0.287	1.946	3.091	3.664

### 5.2 Correlation test

This study conducts the test of the Pearson correlation coefficient matrix for each variable included in the benchmark regression model. According to the results presented in Table 3, the core explanatory variable positively correlates with innovation investment at the 1% significance level, which is consistent with hypothesis 1. In addition, among the paired variables, all the correlation coefficients between DID and control variables are less than 0.5, indicating that there is no serious multicollinearity in the regression model (15).

**Table 3:** Results of correlation test.

Variable	Input	DID	Size	Lev	Cash	Roe	Growth	Shareh10	Age
Input	1								
DID	0.277***	1							
Size	−0.003	0.302***	1						
Lev	−0.087***	0.112***	0.197***	1					
Cash	−0.086***	0.069**	0.048*	−0.308***	1				
Roe	−0.104***	−0.022	0.106***	−0.264***	0.235***	1			
Growth	−0.124***	−0.085***	−0.023	−0.061**	0.044	0.173***	1		
Shareh10	0.023	−0.091***	0.162***	−0.163***	0.163***	0.176***	0.193***	1	
Age	−0.039	0.354***	0.441***	0.206***	−0.095***	−0.025	−0.124***	−0.244***	1

***p < 0.01; **p < 0.05; *p < 0.1.

### 5.3 Baseline empirical results

This section mainly examines the impact of accepting foreign clinical data on the innovation investment of pharmaceutical firms in China. A multi-collinearity test is conducted firstly to avoid the impact of data covariance on the empirical results. As shown in Table 4, the Variance Inflation Factor (VIF) of each variable is less than 10, which indicates there is no multicollinearity in the regression model (15). The benchmark regression is analyzed by gradually adding control variables to Equation 1 with individual and time fixed effects to enhance the reliability of the results. All regressions use robust standard errors, and the estimation results are reported in columns (1) ~ (8) of Table 5. It is observed that the estimated coefficients of the core explanatory variable are all significantly positive regardless of whether control variables are included or not, which indicates that implementing the policy of accepting foreign clinical data significantly stimulates innovation investment of Chinese pharmaceutical firms. The finding is consistent with some previous studies that highlight the positive effect of competition on innovation investment by the escape-competition effect and the technological spillover effect (33). For instance, the study conducted by Guo et al. demonstrated that foreign direct investment (FDI) positively promotes domestic investments in Bangladesh, which may be attributed to the technological spillovers (59).

**Table 4:** Results of covariance test.

Variables	DID	Age	Size	Lev	Shareh10	Roe	Cash	Growth	Mean VIF
VIF	3.73	1.78	1.51	1.29	1.26	1.20	1.20	1.09	1.84

**Table 5:** Baseline empirical results.

Variables	(1)	(2)	(3)	(4)	(5)	(6)	(7)	(8)	(9)	(10)
	Input	Input	Input	Input	Input	Input	Input	Input	Input	Input
DID	2.086***	2.151***	2.145***	2.006***	2.036***	2.090***	2.113***	2.261***	2.504***	1.796***
	(3.03)	(3.04)	(3.04)	(3.21)	(3.34)	(3.41)	(3.47)	(3.63)	(3.07)	(2.94)
Size		−0.707	−0.758	−0.787	−0.361	−0.259	−0.330	−0.157	−1.374	−1.730
		(−0.86)	(−0.90)	(−1.01)	(−0.52)	(−0.37)	(−0.48)	(−0.24)	(−1.33)	(−1.75) *
Lev			0.015	−0.000	−0.020	−0.018	−0.013	−0.017	−0.002	−0.013
			(0.86)	(−0.02)	(−1.42)	(−1.30)	(−0.91)	(−1.16)	(−0.08)	(−0.60)
Cash				−0.180***	−0.141***	−0.138***	−0.134***	−0.125***	−0.156**	−0.146***
				(−3.34)	(−2.96)	(−2.88)	(−2.72)	(−2.73)	(−2.53)	(−2.87)
Roe					−0.086***	−0.075***	−0.076***	−0.079***	−0.065***	−0.063***
					(−4.23)	(−4.02)	(−4.07)	(−4.28)	(−2.88)	(−2.96)
Growth						−0.018***	−0.020***	−0.020***	−0.026**	−0.021**
						(−3.07)	(−3.32)	(−3.28)	(−2.40)	(−2.02)
Shareh10							0.041*	0.054**	0.065*	0.064**
							(1.95)	(2.46)	(1.97)	(2.04)
Age								10.058**	11.752*	3.052
								(2.22)	(1.94)	(0.59)
AC									0.184***	0.176***
									(3.48)	(3.74)
DID×AC										0.380***
										(4.47)
Constant	4.129***	19.513	20.128	22.350	14.320	12.286	11.300	−20.855	−2.285	33.484
	(15.27)	(1.09)	(1.11)	(1.31)	(0.94)	(0.80)	(0.74)	(−1.15)	(−0.09)	(1.26)
Observations	1,248	1,248	1,248	1,248	1,248	1,248	1,248	1,248	1,050	1,050
R-squared	0.7312	0.7325	0.7332	0.7523	0.7691	0.7730	0.7752	0.7800	0.7776	0.8015
Firm FE	YES	YES	YES	YES	YES	YES	YES	YES	YES	YES
Year FE	YES	YES	YES	YES	YES	YES	YES	YES	YES	YES

Robust *t*-statistics in parentheses, ***p < 0.01; **p < 0.05; *p < 0.1.

### 5.4 The moderating effect of absorptive capacity on policy effect

The role of absorptive capacity in promoting innovation was previously held by most scholars (19, 55). According to the theoretical analysis in the previous section, absorptive capacity promotes positively the effect of the accepting foreign clinical data policy on corporate innovation investment. To verify whether this moderating mechanism is valid, we establish the following regression model (51):


(2) $$\begin{array}{l} \text{Inpu}t_{\mathrm{it}}=\alpha +\beta_{1}\text{Trea}t_{\mathrm{it}}\times \mathrm{Pos}t_{\mathrm{it}}+\beta_{2}AC_{\mathrm{it}}+\beta_{3}\text{Trea}t_{\mathrm{it}}\times \\ \;\mathrm{Pos}t_{\mathrm{it}}\times AC_{\mathrm{it}}+\theta X_{\mathrm{it}}+\lambda_{i}+\upsilon_{t}+\varepsilon_{\mathrm{it}} \end{array}$$

In Equation 2, $AC_{\mathrm{it}}$ represents the absorptive capacity of firm i in year t . The empirical results are reported in columns (9) and (10) of Table 5. The results demonstrate that the coefficients of DID are significantly positive, regardless of whether the interaction term DID×AC is included or not, which further confirms hypothesis 2. Furthermore, the coefficient of the interaction term DID×AC is 0.380, which is significantly positive at the 1% significance level. This indicates absorptive capacity positively moderates the influence of the accepting foreign clinical data policy on innovation investment of pharmaceutical firms.

**Figure 3:**
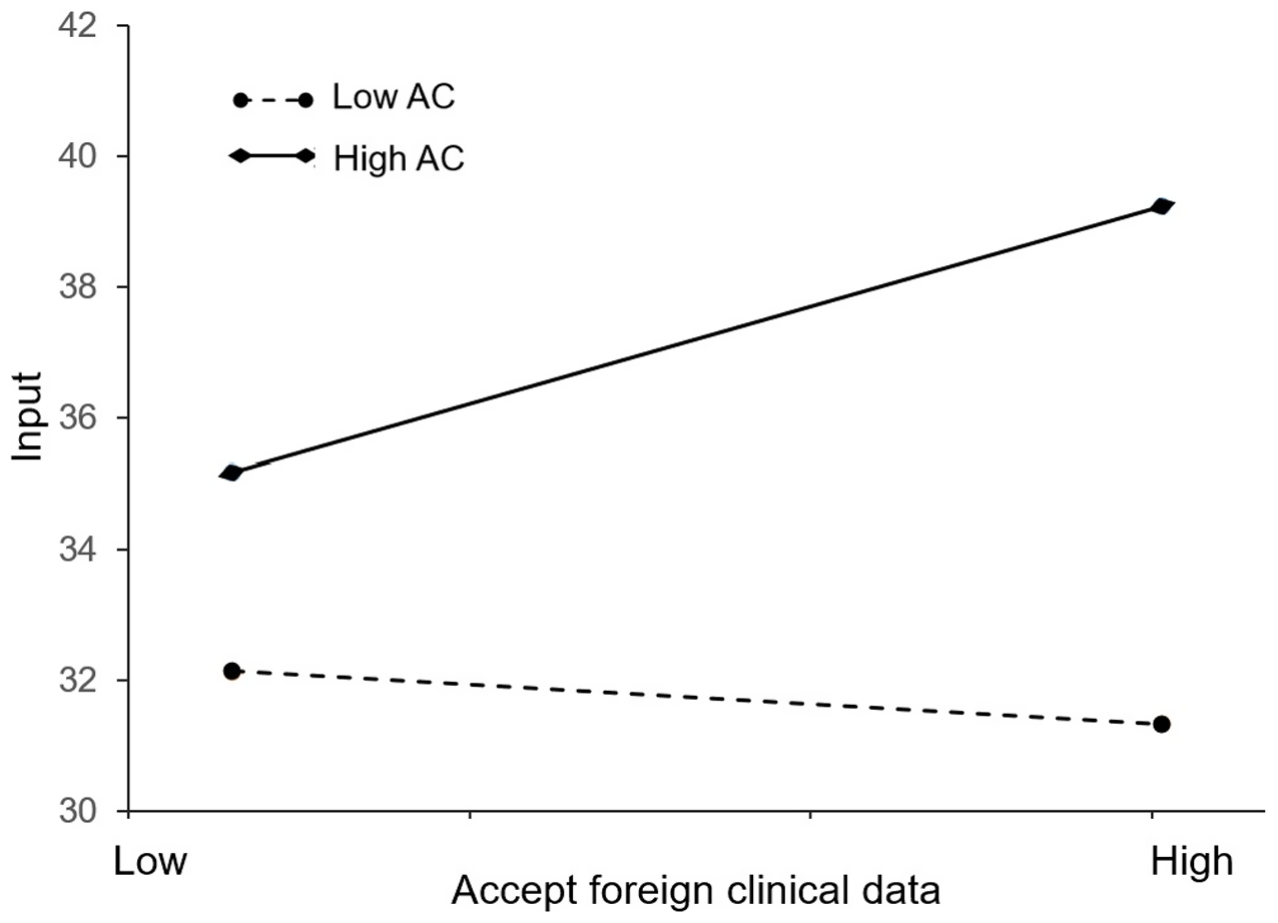
Moderating effect of absorptive capacity on the relationship between the accepting foreign clinical data policy and innovation investment of pharmaceutical firms in China.

Figure 3 is presented to further examine the effect of absorptive capacity. We define high and low on a variable as one standard deviation above or below the mean. One can see that the line with high AC has a positive slope while the line with low AC has a slightly negative slope. This suggests that a firm’s absorptive capacity can enhance the positive effect of the policy of accepting foreign clinical data on its innovation investment. That is, firms with greater absorptive capacity are more likely to increase innovation investment in the face of the policy shock. The finding is consistent with Li and Vanhaverbeke’s study, which has also verified that absorptive capacity may serve as a positive moderator in the relationship between foreign competition and innovation (19). Therefore, hypothesis 2 is supported.

### 5.5 Parallel trend test

The difference-in-differences method is based on the assumption that the explained variable must meet the assumption of parallel trends prior to the implementation of the policy. This means that the innovation investment of both the treatment group and the control group should exhibit a similar changing trend before the samples are affected by the policy. Therefore, drawing on the previous studies (13, 15), this section employs the following model to test the parallel trend:


(3) $${\text{Input}}_{\mathrm{it}}=\alpha +\beta_{k}\sum_{k=-5}^{6}\text{Trea}t_{\mathrm{it}}\times \mathrm{Pos}t_{2018+k}+\theta X_{\mathrm{it}}+\lambda_{i}+\upsilon_{t}+\varepsilon_{\mathrm{it}}$$

**Figure 4:**
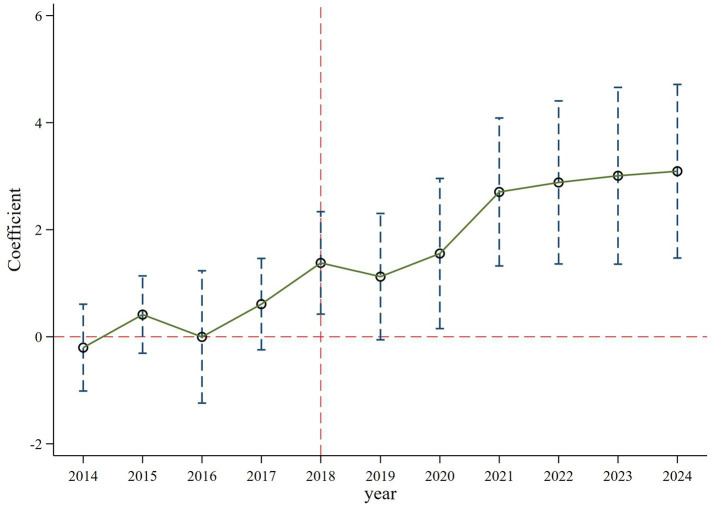
Parallel trend test results with control variables.

In Equation 3, $\mathrm{Pos}t_{2018+k}$ is a dummy variable before and after the policy shock, and k represents time. The estimation result in Figure 4 demonstrates that the confidence interval of all regression coefficients contain 0 and are insignificant within the 90% confidence interval before 2018. Consequently, there is no significant difference in the innovation investment between the treatment and control groups before the implementation of accepting foreign clinical data, suggesting that the selected samples in this study pass the parallel trend test. Further analysis in Table 6 shows that the regression coefficients (coeff2018, coeff2020, coeff2021, coeff2022, coeff2023, coeff2024) after the policy occurred increase and are statistically significant, which implies the positive effect of accepting foreign clinical data on the innovation investment has grown over time (14). This indicates that this policy has a long-term dynamic effect.

**Table 6:** Parallel trend test results.

Variables	Input
coeff2014	−0.203 (−0.42)
coeff2015	0.415 (0.95)
coeff2016	−0.003 (−0.00)
coeff2017	0.609 (1.18)
coeff2018	1.380 ** (2.39)
coeff2019	1.123 (1.58)
coeff2020	1.555* (1.84)
coeff2021	2.705*** (3.25)
coeff2022	2.883*** (3.14)
coeff2023	3.008*** (3.02)
coeff2024	3.093*** (3.17)
Observations	1,248
Control variables	Yes
R-squared	0.7346
Firm FE	YES
Year FE	YES

Robust *t*-statistics in parentheses, ***p < 0.01; **p < 0.05; *p < 0.1.

### 5.6 Robustness tests

#### 5.6.1 Control other policy effect

To improve the approval efficiency of clinical trials and expedite the launch of new drugs, the latest Drug Administration Law in China, carried out on November 1st, 2019, has transitioned the application procedure for drug clinical trials from the prior approval system to an implicit permission system (60). Under this new system, approval will be granted in 60 working days in absence of comment from the Center for Drug Evaluation (CDE). The implicit permission policy for clinical trials also has the potential to stimulate innovation of pharmaceutical firms, which may interfere with our identification for the effect of accepting foreign clinical data. To exclude this potential influence, referring to Li et al. (61), Equation 1 adds the dummy variable $\mathrm{Pos}t_{60}$ indicating whether a pharmaceutical firm applies for clinical trials after 2018 as a control variable for the robustness test. Column (1) of Table 7 presents the estimated results, which demonstrate that the coefficient of DID is 1.704 at the 1% significance level after introducing the dummy variable $\mathrm{Pos}t_{60}$ . This result indicates that the policy of implicit permission for clinical trial applications has little interference with the previous findings, confirming the robustness of our estimated results.

**Table 7:** Robustness test results.

	Input	Input	Input	Input	Input	Input	Input
	(1)	(2)	(3)	(4)	(5)	(6)	(7)
DID	1.704*** (3.18)	0.195** (2.60)	1.188*** (4.78)	2.086*** (3.03)	2.261*** (3.63)	2.059*** (3.14)	2.211*** (3.67)
policy60	1.726*** (2.89)						
Fixed effect	Yes	Yes	Yes	Yes	Yes	Yes	Yes
Control variables	Yes	Yes	Yes	No	Yes	No	Yes

Robust *t*-statistics in parentheses, ***p < 0.01; **p < 0.05; *p < 0.1.

#### 5.6.2 Change the explained variable

Existing studies used the absolute value of R&D expenses or the ratio of R&D expenses to total assets as the explained variable to measure innovation investment (62). To further verify our finding, this study changed the explained variable with these two measures. The results, presented in columns (2) and (3) of Table 7, indicate that the interaction terms are still significantly positive, which are consistent with the previous findings (62).

#### 5.6.3 Placebo test

**Figure 5:**
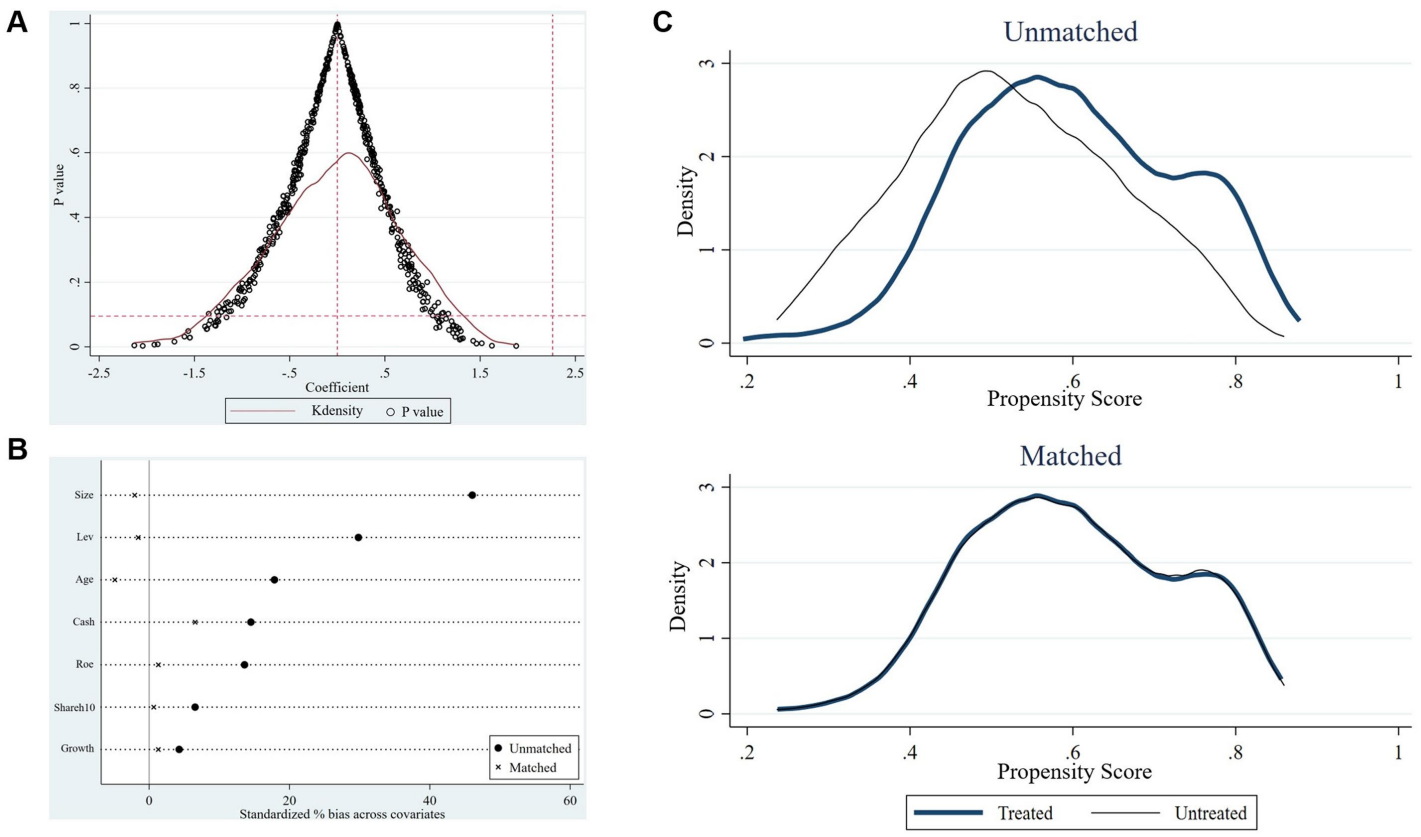
The results of robustness tests. **(A)** The result of the placebo test. **(B)** Standardized deviation before and after matching. **(C)** Propensity score distributions for the treatment and control groups before and after matching.

Tthis study conducts a placebo test to further exclude the influence of other potential unobservable factors on the benchmark regression results. Specifically, we randomly selected the same number of firms from all the samples as the “pseudo-treatment group,” and the remaining firms were set as the control group. Under the condition that the time point of the policy remained unchanged, the regression equation was conducted again to estimate the regression coefficients (14, 15). Since the “pseudo-treatment group” is randomly generated, the regression coefficients of the interaction terms will be insignificant. Otherwise, the benchmark regression results from the policy effect will be spurious. To avoid the interference of small-probability events, this study conducted 500 random samplings (13), and the result is presented in Figure 5A. The figure demonstrates the distribution of the estimated coefficients of the interaction term on the X-axis and the *p* values on the Y-axis. It can be observed that the distribution of the estimated coefficients of the 500 randomly generated “pseudo-policy dummy variables” generally follows the normal distribution with values mainly concentrated around zero, significantly deviating from the actual coefficient estimate of 2.261 (the rightmost vertical dotted line) obtained in model (1). Additionally, the *p* values of a majority of randomly generated coefficients exceed 0.1, indicating these coefficients are statistically insignificant. The above analysis reveals that unobservable random factors do not drive the impact of accepting foreign clinical data on innovation investment. The results of the benchmark regression analysis are consequently robust. This finding confirms the causal relationship between this policy and the explained variable.

#### 5.6.4 PSM-DID test

To reduce the self-selection bias of the estimation results owing to the differences in the characteristics of samples between the treatment group and the control group, this study utilizes the propensity score matching DID method (PSM-DID) to verify the impact of accepting foreign clinical data on the innovation investment of pharmaceutical firms (16). Drawing on the previous study, firms in the treatment group are matched with those in the control group based on the propensity score value, which is achieved by the logit regression with control variables as covariates and the dummy variable Treat as the explained variable (14). Specifically, this study performs a 1:3 caliper nearest-neighbor matching for the control variables between the control and treatment groups, with the caliper value limited to 0.05. Figure 5B and Table 8 show the differences in covariates of control variables before and after matching. The results indicate that the standardized absolute difference of each control variable is significantly reduced to <10% with *p* values greater than 0.1 after matching, and the differences in *t* values between the treatment and control groups are insignificant after matching. Moreover, according to the kernel nuclear density distribution curves of the propensity scores before and after matching in Figure 5C, the difference in the distribution of propensity scores between the treatment and control groups is significantly reduced after matching, suggesting that the data after matching are balanced and the matching is successful. The DID regression results after matching are shown in columns (4) and (5) of Table 7. Furthermore, we also use the kernel matching method and the DID regression results after matching are presented in columns (6) and (7) of Table 7. It is found that the coefficients are still significantly positive, indicating this policy still significantly promotes the innovation investment after matching with two different methods, which is consistent with the original benchmark regression results. The findings confirm the robustness of the benchmark regression results.

**Table 8:** Propensity score matching (PSM) results.

Variable	Matched	Treated	Control	%bias	%reduct |bias|	t	*P*>|t|	V(T)/V(C)
Size	U	22.566	22.118	46.1		8.13	0.000	0.79*
	M	22.556	22.576	−2.1	95.5	−0.40	0.692	0.81*
Lev	U	34.658	29.373	29.8		5.19	0.000	1.19*
	M	34.595	34.871	−1.6	94.8	−0.27	0.784	0.92
Cash	U	6.9855	6.1399	14.5		2.54	0.011	0.95
	M	6.9796	6.5976	6.6	54.8	1.26	0.206	1.01
Roe	U	7.7377	6.1945	13.6		2.37	0.018	1.13
	M	7.7545	7.606	1.3	90.4	0.25	0.800	1.22*
Growth	U	10.573	9.6043	4.3		0.75	0.455	1.09
	M	10.317	10.023	1.3	69.7	0.26	0.798	1.22*
Shareh10	U	53.895	52.92	6.5		1.15	0.249	0.86*
	M	53.782	53.685	0.6	90.1	0.12	0.905	0.82*
Age	U	3.0662	3.0158	17.9		3.14	0.002	0.86*
	M	3.0649	3.0787	−4.9	72.6	−0.96	0.338	1.04

### 5.7 Heterogeneity analysis

The New-New trade theory highlights that firms in the market differ in size, productivity and organizational structure, which means that not all firms can benefit from import liberalization (63). Therefore, the policy effects on the innovation investment of these different types of firms may not be the same. To ensure the accuracy of the benchmark regression results, it is necessary to discuss the heterogeneity effects of the policy on the listed pharmaceutical firms with different ownership patterns, research objects and market power.

#### 5.7.1 Heterogeneous effects on firms with different ownership patterns

Referring to Liu et al. (15), the sample firms are categorized into state-owned and non-state-owned firms based on the classification criteria from the CSMAR. Subsequently, the baseline regression is conducted within these two groups to analyze the heterogeneous effects of the policy on firms with different ownership patterns. The results presented in columns (1) and (2) of Table 9 indicate that the regression coefficient of this policy on innovation investment in non-state-owned firms is significantly higher at 2.456 compared to state-owned firms with a coefficient of only 0.810, which is not statistically significant. Furthermore, a permutation test reveals that the difference in coefficients between these two groups is significant with the *p* value less than 0.01. These findings suggest that the policy of accepting foreign clinical data has a stronger impact on innovation investment among non-state-owned pharmaceutical firms compared to the state-owned firms, which is consistent with the heterogeneous effects of the policies including consistency evaluations of generic drugs, price negotiation in medical insurance, and the Marketing Authorization Holder system in previous studies (13, 15, 17).

**Table 9:** Results of the heterogeneity analysis.

Variables	(1)	(2)	(3)	(4)	(5)	(6)
	State-owned	Non-state-owned	New drug	Non-new drug	Strong market power	Weak market power
	Input	Input	Input	Input	Input	Input
DID	0.810	2.456***	2.805***	−0.076	3.600***	0.385
	(1.40)	(3.14)	(3.71)	(−0.14)	(4.10)	(0.76)
Observations	360	888	804	444	684	540
R-squared	0.7513	0.7790	0.8090	0.7374	0.7994	0.7459
Firm FE	YES	YES	YES	YES	YES	YES
Year FE	YES	YES	YES	YES	YES	YES
Control variables	YES	YES	YES	YES	YES	YES
Permutation test	−1.646***(*p* = 0.006)		−2.882***(*p* = 0.000)		−3.215***(*p* = 0.000)	

The empirical p values of the coefficient differences between the different groups are obtained using the permutation test with 1,000 bootstrap replications. This formula for the Lerner index is as follows: (Operating Revenue - Operating Costs - Sales Expenses - Administrative Expenses) / Operating Revenue × 100%. Robust t-statistics in parentheses, ***p < 0.01; **p < 0.05; *p < 0.1.

The different effects of this policy on the state-owned and non-state-owned firms may be attributed to the variances in their organizational structures and management practices (13). State-owned firms play a dominant role in the Chinese economy, prioritizing the national overall economic stability and strategic security. Their close ties with the government grant them certain non-market privileges in resource acquisition, leading to a relatively lower incentive for technological innovation and a tendency to avoid high-risk innovative activities (16). However, non-state-owned firms, facing less policy preference and greater competitive pressure, are driven to make full use of innovation assets to maximize capital returns. These firms exhibit a higher degree of flexibility in R&D expenses and demonstrate a stronger motivation to acquire innovative technology for enhancing their competitiveness and improve their market position (15). In response to the increased “invasion” of imported drugs due to the policy of accepting foreign clinical data, non-state-owned firms demonstrate a greater ability than state-owned firms to swiftly and effectively identify and respond to market demands, as well as to invest flexibly in innovations.

#### 5.7.2 Heterogeneous effects on firms with different research objects

In this study, listed pharmaceutical firms are divided into the new drug group and the non-new drug group, depending on whether they are applying for clinical trials or marketing authorization for new drugs in 2018 and beyond. The regression results for the two groups are presented in columns (3) and (4) of Table 9. The findings indicate that the accepting foreign clinical data policy has a significant positive effect on innovation investment with a coefficient of 2.805 in the new drug group, while the regression coefficient −0.076 of DID is not statistically significant in the non-new drug group. These results suggest that the accepting foreign clinical data policy can effectively promote innovation investment for firms engaged in new drug research but has no effect on those not involved in new drug research.

The possible reasons are as follows. First of all, while accepting foreign clinical data for new drugs and bioequivalent data for generic drugs expedites the marketing approval process for imported new and generic drugs in China, it is crucial to note that the primary objective of this policy is to facilitate the market entry of innovative drugs (8). According to the viewpoint of signaling theory, this sends a signal to the entire market that the state encourages the R&D of new drugs, thereby motivating pharmaceutical firms to increase investment in researching new drugs (15). Secondly, it is well known that new drug research is characterized by long cycles and high costs (39). The investment required to develop new drugs far exceeds that needed for generic drugs, making the positive effect of this policy on the new drug group more significant than that on the non-new drug group. Finally, Chinese generic drugs have been criticized for overcapacity and homogenization, leading to fierce internal competition among generic drugs (39). Typically, a single type of drug may be produced by numerous domestic firms, rendering the effect of the influx of imported generic drugs on innovation investment among non-new drug firms relatively insignificant.

#### 5.7.3 Heterogeneous effects on firms with different market power

The Lerner index is commonly utilized to measure the market power of firms through their pricing ability in the market (64). The higher the value, the greater the market power of firms within the industry. In this study the samples are categorized into two groups: firms with weak market power and firms with strong market power, based on the median Lerner index in the samples. We report the subsample estimation results for firms with strong and weak market power in columns (5) and (6) of Table 9. The results reveal that the coefficient of DID for strong market power firms is 3.600, which is significant at the 1% level. In contrast, the coefficient 0.385 of DID for weak market power firms is insignificant. The result of the permutation test also suggests that there is a significant difference between the two groups. These results indicate that the policy of accepting foreign clinical data can promote the innovation investment of strong market power firms but has no pronounced effect on weak market power firms. The finding is consistent with Schumpeter’s innovation viewpoint, which posits that firms with substantial monopoly market power have more resources and incentives to innovate (27).

The reason for the differential effects may be that firms with stronger market power have greater capabilities in risk control, overcoming financing constraints, and investing in project innovation (64). This enables them to navigate challenges more effectively and pursue innovative initiatives with greater confidence. In addition, the policy of accepting foreign clinical data facilitates the influx of overseas drugs, especially innovative drugs (8). As competition intensifies from imported drugs alongside the high investment and risks of R&D for new drugs (39), firms with stronger market power are more likely to allocate more resources to the R&D of new drugs in order to maintain their market position.

## 6 Discussion

In this study we evaluated the theoretical predictions concerning the positive effect of the accepting foreign clinical data policy on corporate innovation investment. The results accords with the theories that highlight how import competition promotes domestic innovation activities (32–34). This also corroborates the findings of Shu and Steinwender that the escape-competition effect overwhelmingly exists at firms in developing countries (65). In addition, we also found that corporate absorptive capacity plays a moderating role in the relationship between this policy and innovation investment. This finding is consistent with the previous studies suggesting that adequate absorptive capacity is essential for assimilating external technologies (19, 55). In addition, the heterogeneity effect outcomes of this policy can provide targeted insights for policymakers.

### 6.1 Theoretical implications

This study presents three main theoretical contributions. First, the current literature on the relationship between the policy of accepting foreign clinical data and pharmaceutical firms’ innovation is notably sparse. This paper utilizes a difference-in-differences model to reveal, for the first time, a significantly positive impact of this policy on corporate innovation investment based on the marketing effect and technological effect, which addresses the gap in understanding the relationship between the accepting foreign clinical data policy and innovation. Second, we found that the current literature regarding the impact of imports on innovation primarily focuses on analyzing the effects of import quantity and quality, as well as import tariffs (30, 31, 66). This study contributes to the research on how import-related policies influence firm behavior. Third, this study empirically examines the moderating effect of absorptive capacity and explores the heterogeneity of this policy effect from the perspectives of corporate ownership pattern, research object and market power. This expands the theoretical extension space of the research on corporate innovation investment.

### 6.2 Practical implications

The findings from this study hold important implications for policymakers. In recent years, the Chinese government’s policy-making direction has focused on encouraging the introduction of overseas drugs into the domestic market to address clinical needs (22). Our findings show that the accepting foreign clinical policy significantly promotes the incentives of firms to innovate, which provides valuable evidence and support for policymakers to further improve the relevant policies. Additionally, consistent with the findings of previous studies on policy effects (13, 14), this policy has an insignificant impact on state-owned firms, which gives a hint that more targeted incentive policies should be developed for these firms to improve their innovation incentives. Our study also provides valuable insights for R&D decision-makers in the pharmaceutical sector. The findings regarding both the direct effect and the moderating effect suggest that, in response to the intensifying import competition triggered by this policy, domestic firm should retain their innovation incentives and enhance their absorptive capacities to bolster their competitiveness.

## 7 Conclusions and recommendations

### 7.1 Conclusion

The policy of accepting foreign clinical data is vital to facilitate the marketing of overseas new drugs and improve drug availability for patients in China. Meanwhile, it also enhances the import competition of domestic pharmaceutical firms and improves their access to new drugs. To examine the impact of this policy on the innovation incentives of Chinese pharmaceutical firms and explore the underlying influencing mechanism, this study regards the accepting foreign clinical data policy as a quasi-natural experiment and empirically analyze its implementation effect on pharmaceutical firms’ innovation investment by using the difference-in-differences method. The conclusions are obtained as follows: (1) The policy significantly promotes the innovation investment of Chinese pharmaceutical firms, and the robustness of the empirical results is verified by various methods, including excluding the effect of the implicit permission policy for clinical trial applications, changing the explained variable and utilizing the placebo test method and the PSM-DID method, respectively (2). The absorptive capacity serves as a positive moderator in the relationship between the policy of accepting foreign clinical data and innovation investment of Chinese pharmaceutical firms (3). The policy exhibits heterogeneous impacts on the innovation investment of listed firms due to different ownership patterns, research objects and market power in China, with a significantly positive effect on state-owned firms, new drug firms and strong market power firms, respectively.

### 7.2 Recommendations

Based on the above conclusions, we propose the following policy implications.

Firstly, the accepting foreign clinical data policy accelerates the entry of imported drugs into China, which stimulates the incentives for innovation among Chinese pharmaceutical firms. It is crucial for the drug regulatory authority to learn from the advanced practices of relevant international systems, refine and improve relevant technical requirements, optimize the approval process of clinical trials and speed up the integration of China’s technical guidance system with international general rules in order to promote simultaneous R&D process of overseas new drugs and further accelerate their launch in China.

Secondly, the development of Chinese pharmaceutical innovation industry is currently in a critical transition period. The influx of imported drugs is putting tremendous pressure on domestic new drug firms. In order to mitigate their pressure, It is imperative for the government to provide subsidies, financing support, rewards or technical assistance to firms engaging in R&D for new drugs. These measures will help to reduce their risk and improve their incentive for firms to pursue innovation. Additionally, it is important for the government to take steps to promote the transformation of state-owned firms, stimulate their innovation vitality, and encourage their engagement in innovative activities.

Thirdly, against the backdrop of the accelerating influx of imported drugs into China, it is imperative for Chinese pharmaceutical firms to proactively pursue innovation in order to increase their competitiveness and enhance their resilience against external shocks. On one hand, firms with strong market power should make the most of their own technical experience and resources to drive continuous innovation and facilitate the overall industry’s innovation development. On the other hand, firms with weak market power should increase innovation investment, introduce high-tech personnel and advanced equipment, or engage in collaborative R&D to improve their innovation capability and enhance their market position.

Finally, Chinese pharmaceutical firms should focus on enhancing their absorptive capacity by developing a learning organization and fostering an enterprise culture that values continuous learning. It is recommended to establish an internal knowledge management system, as well as organize various cross-departmental activities for knowledge and information sharing in order to facilitate the diffusion and exchange of knowledge within the organization. Furthermore, it is also crucial to pay attention to building network relationships for acquiring and exchanging external knowledge. Such efforts will contribute to enhancing employees’ abilities to effectively acquire, assimilate and apply new knowledge, ultimately improving their incentives and capabilities to innovate.

## 8 Research limitations and future directions

This study has some limitations that can be further improved. First, this study only includes A-share listed pharmaceutical firms as the research samples, resulting in a relatively small sample size for the benchmark regression. This may introduce potential bias into the conclusions drawn from the analysis. Second, due to data availability constraints, innovation output is not analyzed in this study. It is expected that an increase in innovation investment will drive a corresponding increase in the innovation output of the pharmaceutical industry. Future research on the policy impact on innovation outputs (e.g., number of patent applications, sales of new product) will be conducted once sufficient data is acquired.

## Data Availability

The raw data supporting the conclusions of this article will be made available by the authors, without undue reservation.
